# Chromatin remodeling and epigenetic regulation of mesenchymal stem/stromal cells in osteoarthritis

**DOI:** 10.3389/fgene.2026.1877252

**Published:** 2026-08-07

**Authors:** Mo Wu, Cenzhuo Sheng, Hanhao Zhang, Yuxin Chen, Tianyou Ma, Yirui Tong, Pengchao Xu, Peijian Tong, Hanting Xia

**Affiliations:** 1 Institute of Orthopaedics and Traumatology, The First Affiliated Hospital of Zhejiang Chinese Medical University (Zhejiang Provincial Hospital of Chinese Medicine), Hangzhou, Zhejiang, China; 2 School of Basic Medical Sciences, Liaoning University of Traditional Chinese Medicine, Shenyang, Liaoning, China

**Keywords:** chondrogenesis, chromatin remodeling, epigenetics, extracellular vesicles, mesenchymal stem/stromal cells, osteoarthritis, regenerative medicine

## Abstract

Osteoarthritis (OA) creates a persistently hostile joint microenvironment in which inflammatory, mechanical, metabolic, oxidative, and senescence-related cues alter the behavior of mesenchymal stem/stromal cells (MSCs). These changes are not limited to short-term signaling responses, but they should not be interpreted as fixed or irreversible cellular states. Depending on the duration and intensity of environmental exposure, some MSC responses may be transient and reversible, whereas others may be reinforced through epigenetic and chromatin-state regulation, potentially contributing to impaired chondrogenic differentiation, accelerated cellular senescence, and altered paracrine activity, including extracellular vesicles (EVs)-related functions. In this review, we discuss how DNA methylation, histone modifications, long non-coding RNA (lncRNA)-mediated complex recruitment and RNA modifications, chromatin accessibility, and adenosine triphosphate (ATP)-dependent chromatin remodeling connect OA-related stress with OA-associated functional alterations of MSCs. Particular attention is given to the position of chromatin remodeling within the broader epigenetic network. Rather than acting as a separate mechanism, chromatin remodeling provides a structural layer through which regulatory marks, enhancer activity, nucleosome positioning, and transcription-factor access are translated into transcriptional outcomes. We also distinguish different levels of evidence. Data linking epigenetic regulation to MSC chondrogenesis are relatively strong, whereas direct evidence that specific chromatin events determine cargo loading into EVs remains limited. Chromatin regulators such as histone deacetylases (HDACs), BRG1/SMARCA4, and SMARCA5 may serve as mechanistic entry points, but findings from chondrocytes or general MSC models should not be directly extrapolated to OA-derived MSCs without further validation. Finally, we discuss rejuvenation of MSCs, epigenetic preconditioning, microenvironmental engineering, and optimization of EVs as potential strategies for OA regenerative therapy, while emphasizing product heterogeneity, potency testing, patient stratification, and long-term safety as major barriers to translation.

## 1 Introduction

Osteoarthritis (OA) is a chronic whole-joint disorder rather than a simple consequence of articular cartilage wear. Its pathological changes involve cartilage degradation, subchondral bone remodeling, synovitis, and alterations in other joint tissues, which together contribute to pain, stiffness, and functional limitation (Hunter and Bierma-Zeinstra, 2019; Katz et al., 2021). This whole-joint view has changed how OA progression is interpreted: increased or altered mechanical loading, low-grade inflammation, and crosstalk among cartilage, synovium, subchondral bone, and other joint compartments are now considered central contributors to disease development and progression (Loeser et al., 2012; Sheng et al., 2025; Hunter and Bierma-Zeinstra, 2019; Roelofs and De Bari, 2024; Zhou et al., 2025). This local whole-joint concept can be further extended to a broader skeletal-pathophysiology perspective, in which endocrine, immune, metabolic, and neural interorgan communications may influence bone and joint homeostasis and disease progression (Gan et al., 2026).

Mesenchymal stem/stromal cells (MSCs) are relevant to OA repair because they can self-renew, differentiate into multiple lineages, regulate immune responses, and secrete paracrine factors. Unless otherwise specified, the mechanistic sections of this review primarily focus on endogenous or tissue-resident MSC populations within OA joint tissues, including synovial MSCs, bone marrow-derived MSCs, and other local stromal/progenitor cell populations. Therapeutically administered MSCs and MSC-derived extracellular vesicles (EVs) are discussed separately in the translational and regenerative-therapy sections. Their therapeutic effects in OA, however, are unlikely to depend mainly on direct replacement of lost chondrocytes. Current evidence more strongly supports a paracrine and microenvironment-modifying role, in which MSCs release growth factors, immunomodulatory molecules, and extracellular vesicles (EVs) that can influence inflammation, matrix metabolism, and repair-related cellular communication. This potential is constrained by the OA joint environment. Persistent inflammatory stimulation, increased or altered mechanical loading, and tissue damage may reduce survival, homing, and differentiation stability of MSCs, thereby limiting their repair effect *in vivo* (Eseonu and De Bari, 2015; Molnar et al., 2022).

A key problem is that OA-associated functional alterations of MSCs may extend beyond acute signaling responses, while remaining dynamic and context-dependent. Under physiological conditions, MSCs are characterized by self-renewal capacity, multilineage differentiation potential, immunomodulatory activity, and paracrine support of tissue repair. In OA, prolonged exposure to a pathological microenvironment may shift MSCs toward a repair-unfavorable functional state characterized by reduced proliferative capacity, impaired chondrogenic differentiation, increased senescence-associated features, altered immunomodulatory activity, and changes in paracrine function. However, this state may reflect cellular adaptation to changing environmental cues rather than a fixed loss of MSC identity (Liu et al., 2020; Smith et al., 2023).

Acute signaling responses generally occur within minutes to hours, whereas changes in cellular activation status, differentiation behavior, retention, and paracrine function may develop over days. Epigenetic and chromatin-state regulation are more commonly associated with sustained pathological exposure over longer time scales. DNA methylation, histone modifications, chromatin-state changes, and non-coding RNA regulation may help reinforce disease-associated transcriptional programs during sustained exposure, although some of these changes may remain reversible when the microenvironment changes (Wang et al., 2020).

Importantly, these OA-associated functional alterations should not be interpreted as fixed or irreversible cellular states. MSCs continuously adapt to changing microenvironmental conditions, and some transcriptional, epigenetic, and functional changes may be transient, reversible, and context-dependent. Therefore, the functional phenotype of MSCs likely reflects a dynamic equilibrium between environmental stimuli and cellular adaptation rather than a permanently altered cellular identity.

In this review, epigenetic regulation refers to an integrated regulatory system that includes DNA methylation, histone modifications, non-coding RNAs, RNA modifications, and chromatin remodeling. These layers do not act independently. DNA methylation and histone modifications provide regulatory marks; non-coding RNAs can participate in regulatory-complex recruitment and transcriptional control; RNA modifications may influence post-transcriptional regulation and fate-related gene-expression programs; and chromatin remodeling changes nucleosome positioning, chromatin conformation, and regulatory-element accessibility through adenosine triphosphate (ATP)-dependent remodeling complexes (Centore et al., 2020; Eustermann et al., 2024; Chen et al., 2024). From this perspective, chromatin remodeling should not be treated as separate from epigenetic regulation. It is better understood as a structural layer that links microenvironmental stress, epigenetic mark changes, enhancer activity, and functional reprogramming of MSCs.

Several molecular nodes illustrate this connection. BRG1/SMARCA4 and SMARCA5 are ATP-dependent chromatin remodeling factors, whereas histone deacetylases (HDACs) regulate histone acetylation status and chromatin accessibility through deacetylation-related mechanisms. Together, these molecules provide useful entry points for understanding how OA-related stimuli may become translated into sustained but potentially reversible chromatin-state alterations (Alessio et al., 2010; Zhang et al., 2020; Mao et al., 2023; Xu et al., 2025). However, the evidence should be interpreted cautiously. Some findings come from chondrocytes or general MSC senescence models rather than OA-derived MSCs, so they cannot be directly extrapolated to all aspects of MSC chondrogenesis, senescence, or EV-related function in OA.

**FIGURE 1:**
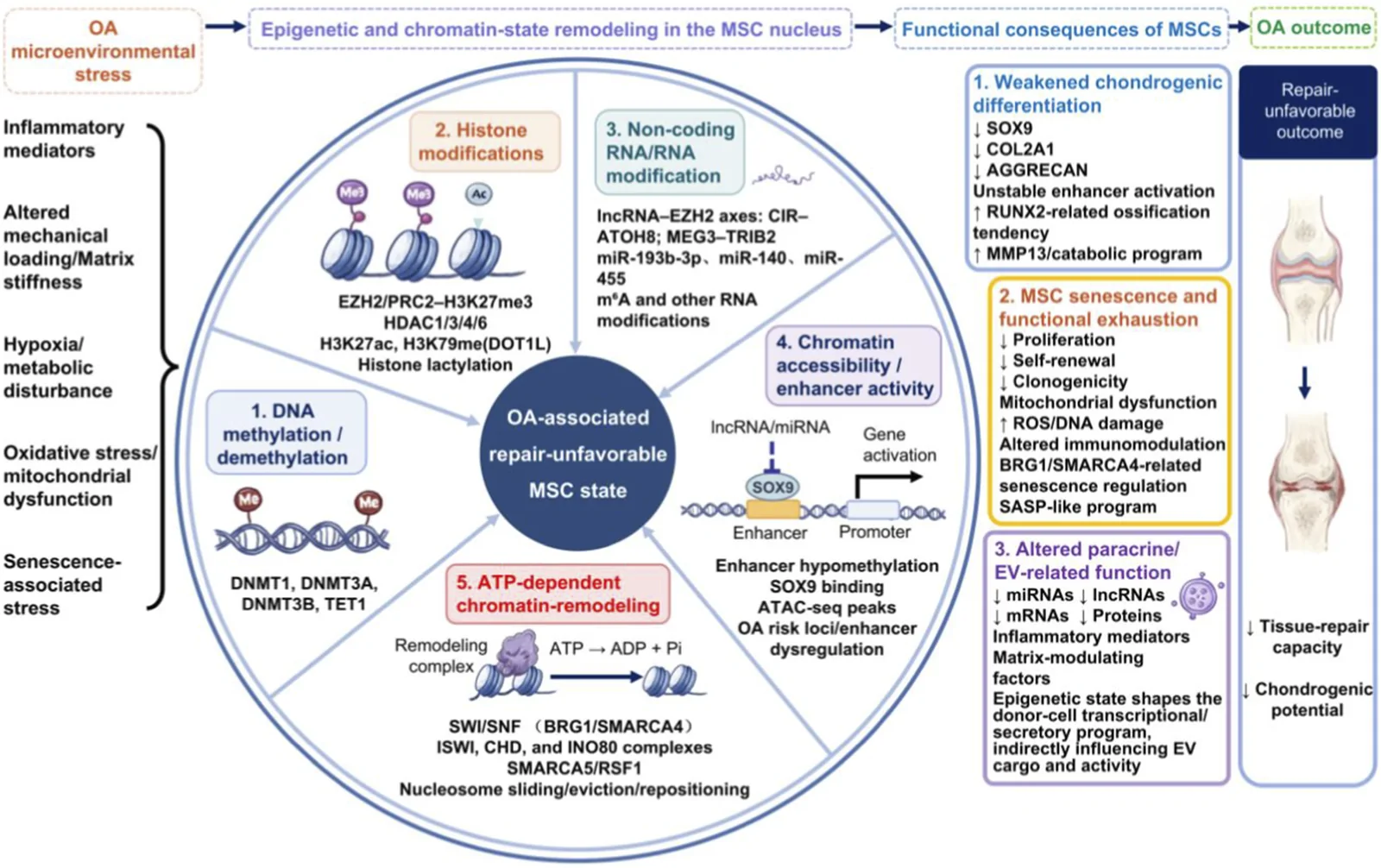
Epigenetic and chromatin-state regulation of MSCs in the osteoarthritis microenvironment. Inflammatory factors, increased or altered mechanical loading, hypoxia, metabolic disturbance, and senescence-associated stress in the osteoarthritis (OA) microenvironment can jointly act on the epigenetic regulatory network of mesenchymal stem/stromal cells (MSCs). This network includes DNA methylation, histone modifications, non-coding RNA/RNA modifications, and chromatin remodeling. Chromatin remodeling is mainly reflected in changes in chromatin structure, nucleosome positioning, regulatory-element accessibility, and enhancer activity, and can be modulated by DNA methylation, histone acetylation/methylation, and long non-coding RNA (lncRNA)-mediated complex recruitment. These changes further affect chondrogenic capacity, senescence and functional exhaustion, and paracrine/extracellular vesicle (EV)-related function of MSCs, ultimately contributing to impaired tissue repair and OA progression.

The central question of this review is how OA-related microenvironmental stress becomes embedded in MSC regulatory programs. To address this question, we link inflammatory cues, increased or altered mechanical loading, hypoxia/metabolic disturbance, and senescence-associated stress with DNA methylation, histone modifications, non-coding RNA networks, RNA modifications, chromatin accessibility, nuclear architecture, and ATP-dependent remodeling. We then discuss how these regulatory changes may influence chondrogenic differentiation, cellular senescence, paracrine activity, and EV-related repair activity. By distinguishing evidence derived from endogenous OA-associated MSCs, therapeutically administered MSCs, chondrocytes, and general MSC models, this review aims to clarify which epigenetic and chromatin-state mechanisms are most relevant to OA-associated functional alterations of MSCs and which may be suitable for future regenerative strategies (Figure 1).

## 2 Epigenetic and chromatin-state responses of MSCs to the OA microenvironment

The OA microenvironment influences MSCs through more than transient inflammatory activation. Persistent exposure to inflammatory mediators, increased mechanical loading, extracellular matrix changes, hypoxia, metabolic disturbance, oxidative stress, and senescence-associated signals can gradually shift the regulatory state of MSCs. The OA microenvironment influences MSCs through more than transient inflammatory activation. Persistent exposure to inflammatory mediators, increased or altered mechanical loading, extracellular matrix changes, hypoxia, metabolic disturbance, oxidative stress, and senescence-associated signals can gradually shift the regulatory state of MSCs. These stimuli may converge on transcriptional programs and multiple epigenetic regulatory layers, including DNA methylation, histone acetylation/methylation, non-coding RNAs, RNA modifications such as N6-methyladenosine (m6A), chromatin accessibility, enhancer activity, nucleosome positioning, and ATP-dependent chromatin remodeling. The resulting changes may alter differentiation potential, senescence progression, and paracrine function in MSCs (Zhang Q. et al., 2024; Walewska et al., 2023; Wang et al., 2020; Liu et al., 2018; Barter et al., 2020; Cheung et al., 2020; Centore et al., 2020; Eustermann et al., 2024; Chen et al., 2024). To understand these effects, it may be useful to view OA-related stimuli not as isolated triggers, but as a series of interacting stresses that progressively reshape the chromatin and epigenetic state of MSCs.

Inflammation is one of the clearest examples of this process. Interleukin-1β (IL-1β) and tumor necrosis factor-α (TNF-α) can suppress chondrogenic differentiation of MSCs, interfere with SOX9-dependent cartilage programs, and activate catabolic or stress-related pathways, thereby weakening cartilage-repair potential (Kondo et al., 2014). For example, IL-1β exposure reduces the expression of chondrogenic markers such as SOX9 and COL2A1, whereas TNF-α suppresses cartilage matrix formation and impairs TGF-β-mediated chondrogenesis through activation of inflammatory signaling pathways including NF-κB (Wehling et al., 2009; Kondo et al., 2014). The significance of inflammatory exposure, however, is not limited to short-lived transcriptional changes. Inflammatory signaling may also influence DNA methylation, histone modifications, non-coding RNAs, and chromatin regulatory processes, which helps explain why OA-associated functional alterations of MSCs may be maintained or reinforced during chronic stimulation, while remaining context-dependent (Walewska et al., 2023). Recent evidence in knee OA further suggests that HMGB1 can promote IRF1 SUMOylation through MyD88, thereby priming trained immunity in circulating monocytes and aggravating progressive synovial inflammation (Jie et al., 2026). Although this evidence concerns monocytes rather than MSCs, it reinforces the concept that chronic OA inflammation may be sustained through immune-cell priming and inflammatory feedback. At the chromatin level, the HDAC6/nuclear factor-κB (NF-κB) axis has been linked to inflammatory matrix metalloproteinases (MMPs) and inflammatory gene expression, whereas HDAC3 regulates acetylation at cartilage-specific gene loci. These findings suggest that distinct HDAC subtypes may provide routes through which inflammatory signals enter chromatin-level regulation (Meng et al., 2018; Barter et al., 2022).

Mechanical stress provides another route by which the OA microenvironment may influence MSC fate. Altered joint loading, extracellular matrix composition, and matrix stiffness can act through integrins, focal adhesions, the cytoskeleton, and nuclear mechanotransduction to affect transcriptional activity and lineage direction in MSCs (Steward and Kelly, 2015; Saidova and Vorobjev, 2020). Direct evidence from OA-derived MSCs remains limited, but mechanobiology studies support the idea that mechanical inputs can be translated into nuclear and chromatin-level changes. Mechanical loading can induce nuclear deformation, chromatin condensation, and reorganization of nuclear architecture, thereby influencing lineage commitment and transcriptional activity in MSCs. Increased substrate stiffness has been shown to promote Lamin A/C accumulation and heterochromatin formation, whereas mechanical stimulation can alter nuclear morphology and chromatin organization through cytoskeleton–nucleus coupling mechanisms (Heo et al., 2016; Ankam et al., 2018). Furthermore, mechanical stimulation can affect differentiation pathways in MSCs through HDAC1 and related chromatin states, indicating that mechanical signals may act not only through membrane and cytoplasmic pathways but also through nuclear architecture, nucleosome organization, and deacetylation-related epigenetic homeostasis (Wang et al., 2016). In OA, increased mechanical loading may therefore serve as an upstream driver linking joint biomechanical imbalance with altered chromatin states in MSCs.

Hypoxia and metabolic disturbance add another layer to this regulatory network. Moderate hypoxia can elevate some cartilage-associated markers and support stemness under specific culture conditions, but this effect depends strongly on oxygen tension, culture stage, cell origin, and induction protocol; therefore, hypoxia should not be treated as uniformly pro-chondrogenic (Ranmuthu et al., 2022). Hypoxia can also induce metabolic reprogramming in MSCs, affecting survival, differentiation capacity, and paracrine activity. In the OA joint, hypoxia is unlikely to act as a single variable. It may interact with inflammation and nutritional stress to shape MSC fate (Beegle et al., 2015; Walewska et al., 2023). Mechanistically, metabolic shifts can change the availability of epigenetic substrates and cofactors, including S-adenosylmethionine (SAM), acetyl-CoA, nicotinamide adenine dinucleotide (NAD+), and lactate. These metabolites may influence DNA and histone methylation, histone acetylation, histone lactylation, and chromatin opening, thereby linking metabolic state with transcriptional regulation in MSCs (Lu and Thompson, 2012; Etchegaray and Mostoslavsky, 2016; Walewska et al., 2023; Zhao et al., 2025). For example, reduced NAD+ availability can decrease sirtuin activity and impair mitochondrial homeostasis, whereas increased glycolytic flux and lactate accumulation may promote histone lactylation and alter the expression of inflammation- and metabolism-related genes (Etchegaray and Mostoslavsky, 2016; Zhang et al., 2019; Zhao et al., 2025). Consistent with this metabolism–epigenetics connection, evidence from intervertebral disc degeneration, another degenerative musculoskeletal disorder, indicates that metabolism- and inflammation-related post-translational modifications, including phosphorylation, ubiquitination, acetylation, glycosylation, methylation, and lactylation, can reshape tissue phenotype and disease progression (Zhu et al., 2024). However, these findings should be used as supportive cross-disease evidence rather than direct evidence for OA-derived MSCs.

Recent evidence from OA chondrocytes further supports a connection between metabolism and chromatin regulation. The SMARCA5/RSF1 complex can bind glycolysis-related gene promoters and increase histone H3 lysine 27 acetylation (H3K27ac) and transcriptional activity through an ATPase-dependent mechanism, thereby promoting pathological glycolysis and chondrocyte dysfunction in OA (Xu et al., 2025). This finding is mechanistically relevant for understanding OA-related metabolic remodeling, but it should not be directly extrapolated to OA-derived MSCs. Whether similar SMARCA5/RSF1-dependent chromatin regulation operates in MSCs remains an open question.

Senescence-associated stress in the OA joint may originate from both MSC-intrinsic changes and senescence-related signals released by surrounding joint-resident cells. Senescent chondrocytes, synoviocytes, and other OA-associated cells can generate senescence-associated secretory phenotype (SASP) factors that influence MSC behavior. At the same time, aging and continuous pathological stimulation may increase the susceptibility of MSCs themselves to DNA damage accumulation, elevated reactive oxygen species (ROS), mitochondrial dysfunction, and senescence-associated phenotypes (Cheng et al., 2023; Smith et al., 2023).

Importantly, MSC quiescence should not be equated with cellular senescence. Because tissue-resident MSCs may naturally remain in a relatively quiescent state, reduced proliferation or low activation alone should not be interpreted as senescence. Some senescence-like phenotypes in OA-associated MSCs may instead reflect insufficient activation or altered responsiveness to the pathological microenvironment.

These pathways are best understood as interacting cycles rather than separate mechanisms. Mechanical loading and extracellular matrix remodeling may act relatively upstream by altering nuclear structure, chromatin accessibility, and the responsiveness of MSCs to inflammatory signals (Steward and Kelly, 2015; Saidova and Vorobjev, 2020; Wang et al., 2016). Inflammatory mediators can then amplify stress-related transcriptional responses through NF-κB-related pathways and HDAC-mediated chromatin regulation, while also promoting oxidative stress and mitochondrial dysfunction (Kondo et al., 2014; Meng et al., 2018; Barter et al., 2022; Walewska et al., 2023). In parallel, hypoxia and metabolic disturbance may act both as consequences of the pathological microenvironment and as active regulators of epigenetic remodeling by changing the availability of metabolites required for methylation, acetylation, deacetylation, and lactylation (Beegle et al., 2015; Lu and Thompson, 2012; Etchegaray and Mostoslavsky, 2016; Ranmuthu et al., 2022; Zhao et al., 2025). Senescence may further stabilize these changes and reinforce local inflammation, oxidative stress, mitochondrial dysfunction, and catabolic signaling (Cheng et al., 2023; Smith et al., 2023; Walewska et al., 2023).

From this perspective, OA-related altered functional state of MSCs may be maintained by positive-feedback interactions among mechanical stress, inflammation, metabolic reprogramming, and senescence. Chromatin remodeling acts as an integrative layer within this process by linking DNA methylation, histone modifications, non-coding RNA/RNA modifications, and metabolic inputs to nucleosome positioning, regulatory-element accessibility, enhancer activity, and transcription-factor binding. These changes may convert repeated microenvironmental stimulation into reinforced but potentially reversible transcriptional and functional alterations, leading to impaired proliferation, altered differentiation direction, reduced paracrine activity, and weakened cartilage-repair capacity. The duration and magnitude of these responses may vary according to the intensity and persistence of environmental stimuli, indicating that MSC functional states remain dynamic and context-dependent rather than permanently fixed (Wang et al., 2020; Liu et al., 2018; Barter et al., 2020; Cheung et al., 2020; Centore et al., 2020; Eustermann et al., 2024; Chen et al., 2024; Zhang Q. et al., 2024) (Table 1).

**TABLE 1:** OA microenvironmental factors associated with epigenetic and chromatin-state alterations in MSCs.

OA microenvironmental factor	Representative molecular events	Potential epigenetic/chromatin-state alterations	Potential effects on MSCs	Representative references
Inflammatory mediators (IL-1β, TNF-α)	IL-1β ↓ SOX9/COL2A1; TNF-α → NF-κB activation	DNA methylation, histone modifications, HDAC-mediated chromatin regulation	Reduced chondrogenesis, altered immunomodulation, enhanced inflammatory responsiveness	Wehling et al. (2009); Kondo et al. (2014); Meng et al. (2018); Barter et al. (2022)
Increased or altered mechanical loading/matrix stiffness	Nuclear deformation; Lamin A/C accumulation; heterochromatin formation	Nuclear architectural remodeling, chromatin condensation, altered accessibility	Altered lineage commitment and impaired repair capacity	Steward and Kelly (2015); Saidova and Vorobjev (2020); Heo et al. (2016); Ankam et al. (2018); Wang et al. (2016)
Hypoxia and metabolic disturbance	NAD+ depletion → reduced sirtuin activity; lactate accumulation → histone lactylation	Altered methylation, acetylation, deacetylation, and lactylation states	Altered stemness maintenance, metabolism, differentiation, and paracrine activity	Beegle et al. (2015); Ranmuthu et al. (2022); Etchegaray and Mostoslavsky (2016); Zhang et al. (2019); Zhao et al. (2025)
Oxidative stress/mitochondrial dysfunction	ROS accumulation and DNA damage responses	Stress-associated epigenetic remodeling and chromatin instability	Increased senescence-associated features and reduced repair capacity	Cheng et al. (2023); Smith et al. (2023)
Senescence-associated stress/SASP exposure	SASP factors from senescent chondrocytes and synoviocytes	Reinforcement of senescence-related transcriptional programs	Reduced proliferation, clonogenicity, and chondrogenic potential	Huang et al. (2020); Miura et al. (2022); Teti et al. (2023)

## 3 Epigenetic regulation and weakened chondrogenic differentiation of MSCs

### 3.1 DNA methylation

DNA methylation provides a relatively stable but context-dependent layer of epigenetic regulation in MSCs. In early models, it was often discussed mainly in relation to promoter silencing and transcriptional repression. For chondrogenic differentiation of MSCs, however, this promoter-centered view is too narrow. Current evidence indicates that DNA methylation changes are closely linked to enhancer activity, transcription-factor binding, and local chromatin accessibility, suggesting that chondrogenesis depends on coordinated regulation across multiple cis-regulatory elements rather than on individual cartilage-gene promoters alone (Barter et al., 2020; Cheung et al., 2020).

During normal chondrogenic differentiation, DNA methylation changes occur together with shifts in histone marks, enhancer activation, and transcription-factor occupancy. Genome-wide analyses have shown that DNA hypomethylation during differentiation of MSCs toward the chondrogenic lineage is enriched predominantly at enhancer regions. These enhancer-associated methylation changes are accompanied by dynamic histone modification patterns related to cartilage formation, indicating that DNA methylation helps determine not only whether individual genes are expressed but also whether regulatory elements become permissive for a chondrogenic transcriptional program (Herlofsen et al., 2013; Barter et al., 2020). The close association between enhancer activation and SOX9 enrichment further supports this coordinated model of DNA methylation, enhancer state, and cartilage-related gene expression (Cheung et al., 2020).

In OA, this regulatory coordination may be disrupted. Age-related changes, inflammatory stimulation, increased or altered mechanical loading, and metabolic perturbation can reshape DNA methylation patterns and thereby interfere with chondrogenic programs in MSCs. Evidence from OA articular cartilage shows that differentially accessible chromatin regions are frequently enriched at enhancers and are associated with abnormal activation of ossification- and MSCs differentiation-related pathways (Liu et al., 2018). Although these findings are not all derived directly from OA-derived MSCs, they are relevant because they point to enhancer-rich regulatory regions as potential sites where disease-associated epigenetic instability may occur.

A cautious interpretation is therefore needed. Impaired chondrogenesis of MSCs in OA should not be explained only by methylation changes at single cartilage-gene promoters. It is more likely to involve a broader regulatory disturbance in which abnormal DNA methylation interacts with histone modifications, enhancer activity, chromatin accessibility, and transcription-factor binding. Such changes may make it more difficult for MSCs to maintain a stable chondrogenic fate and may increase the tendency toward fibrotic, hypertrophic, or ossification-related differentiation states.

### 3.2 Histone modifications and long non-coding RNA (lncRNA)-mediated regulation

Histone modifications provide a more dynamic regulatory layer than DNA methylation in the fate control of MSCs. Methylation, acetylation, and other histone marks can modify local chromatin structure by changing histone-DNA interactions and by recruiting reader proteins or regulatory complexes. In OA-associated MSCs, this layer is particularly relevant because repressive histone states, enhancer-associated histone activity, and HDAC-mediated deacetylation may all influence chondrogenic differentiation, inflammatory responsiveness, and cellular functional decline (Ren et al., 2020; Walewska et al., 2023; Zhang et al., 2020).

One important example is enhancer of zeste homolog 2 (EZH2), the catalytic subunit of polycomb repressive complex 2 (PRC2). EZH2 mediates histone H3 lysine 27 trimethylation (H3K27me3), a repressive histone mark involved in lineage regulation of MSCs (Margueron and Reinberg, 2011; Liu et al., 2021). During chondrogenic differentiation of MSCs, activation of cartilage-related genes is usually accompanied by reduced H3K27me3-mediated repression and increased active histone marks. If H3K27me3-associated repression persists or becomes abnormally enhanced, progression of the chondrogenic program may be constrained, making EZH2-related regulation a potential epigenetic node in altered differentiation of MSCs (Ren et al., 2020; Walewska et al., 2023).

Long non-coding RNAs (lncRNAs) add another level of specificity to this process by helping recruit chromatin-modifying complexes to particular genomic regions. In human umbilical cord-derived MSCs (hUC-MSCs), lncRNA *CIR* can bind EZH2 and promote EZH2-mediated H3K27me3 enrichment at the *ATOH8* promoter, thereby suppressing *ATOH8* expression and impairing chondrogenic differentiation. This *CIR*–EZH2–*ATOH8* axis provides relatively direct evidence that lncRNA-guided recruitment of EZH2 can inhibit chondrogenesis (Liu et al., 2021). More broadly, interactions between lncRNAs and EZH2 or other chromatin-modifying complexes are increasingly recognized as mechanisms through which fate decisions of MSCs may be altered in degenerative bone and joint diseases (Xia et al., 2022).

Beyond methylation-related histone marks, histone acetylation and deacetylation also participate in the regulation of MSC function. Acetylated histones are generally associated with chromatin regions that are more permissive to transcriptional activation. HDACs counter this process by removing acetyl groups, thereby influencing proliferation, differentiation, and senescence-related phenotypes of MSCs. In this sense, HDACs can be viewed as a regulatory axis linking histone modification, chromatin-state change, and functional remodeling of MSCs (Jang et al., 2022; Walewska et al., 2023; Zhang et al., 2020).

Histone lactylation further connects metabolic state with histone modification. Under hypoxic conditions, enhanced glycolysis and lactate accumulation may allow lactate to act not only as a metabolic end-product but also as a substrate for lactylation, thereby contributing to the regulation of gene expression and function in MSCs. However, direct evidence for lactylation in OA-related chondrogenic differentiation of MSCs remains limited. It is therefore more appropriate to treat lactylation as an emerging mechanism that may link hypoxia, metabolism, and epigenetic regulation, rather than as an established driver of OA-related altered functional state of MSCs (Zhao et al., 2025).

Different HDAC subtypes should also be distinguished because their functions are not interchangeable. HDAC1 has been implicated in transforming growth factor-β1 (TGF-β1)-mediated early chondrogenic differentiation. HDAC3 is associated with repression of cartilage-specific genes, whereas the microRNA-193b-3p (miR-193b-3p)/HDAC3 axis can enhance chondrogenic differentiation in human MSCs (hMSCs) by increasing H3 acetylation at the *COL2A1*, *AGGRECAN*, and *SOX9* loci, while also improving the catabolic phenotype of OA chondrocytes. HDAC6 participates in interleukin-1β (IL-1β)-induced nuclear factor-κB (NF-κB) signaling, matrix metalloproteinases (MMPs), and inflammatory gene expression. HDAC4 appears to protect OA cartilage by suppressing the activating transcription factor 4 (ATF4)–CCAAT/enhancer-binding protein homologous protein (CHOP) axis, which is linked to endoplasmic reticulum (ER) stress and apoptosis, thereby slowing cartilage degeneration (Huang et al., 2014; Meng et al., 2018; Zhang et al., 2020; Barter et al., 2022; Gu et al., 2024). These subtype-specific findings suggest that HDACs should not be discussed as a single uniform target in OA-related regulation of MSCs. Collectively, these studies further illustrate that histone-modifying enzymes do not merely alter global chromatin states, but can directly regulate key chondrogenic genes and pathways, including SOX9, COL2A1, and ACAN/AGGRECAN, thereby influencing lineage commitment, cartilage matrix formation, and functional remodeling of MSCs.

### 3.3 Chromatin accessibility, enhancer changes, and ATP-dependent chromatin remodeling

Chromatin accessibility determines whether regulatory DNA regions can be reached by transcription factors and the transcriptional machinery (Chen et al., 2024). In MSCs, this feature is especially important during chondrogenesis because cartilage-related transcription does not depend only on promoter activation. Instead, enhancer activation, local chromatin opening, and nucleosome repositioning cooperate to create regulatory conditions that permit cartilage-gene expression (Cheung et al., 2020; Barter et al., 2026; Eustermann et al., 2024). During chondrogenic differentiation, chromatin regions that were previously inaccessible can become open. Many of these regions are located near cartilage-associated enhancers and contain binding motifs for SOX9 and other key transcription factors. These chromatin changes often occur together with DNA hypomethylation and active enhancer-associated histone marks, indicating that chondrogenesis of MSCs depends on coordinated remodeling across multiple regulatory elements rather than on a single promoter-centered program (Herlofsen et al., 2013; Barter et al., 2020; Cheung et al., 2020; Barter et al., 2026).

Enhancers provide a regulatory interface between external cues and endogenous transcriptional programs. Under physiological conditions, cartilage-associated enhancers gain activity as nearby chromatin becomes more accessible, supporting sustained cartilage-gene expression and stabilization of the chondrogenic phenotype (Barter et al., 2020; Herlofsen et al., 2013; Cheung et al., 2020). In the OA microenvironment, this enhancer-centered regulation may become unstable. Inflammation, senescence, increased or altered mechanical loading, and metabolic disturbance can interfere with normal enhancer activation and shift transcriptional networks in MSCs away from stable chondrogenesis. Evidence from OA tissues shows that aberrantly accessible chromatin regions are enriched at enhancers and are associated with ossification- and MSCs differentiation-related pathways (Liu et al., 2018). These findings suggest that OA-related epigenetic imbalance may allow MSCs to express some cartilage-associated molecules without fully establishing a stable chondrogenic phenotype (Walewska et al., 2023).

This enhancer-based perspective is also useful for interpreting OA-associated non-coding genetic variants. Many OA risk loci are located outside protein-coding regions and overlap with enhancers or other accessible chromatin regions. Their biological effects may therefore arise not from altered protein sequence but from changes in enhancer activity, transcription-factor binding, or local chromatin accessibility. Compared with assigning each non-coding variant to a distant target gene, integrating enhancer annotation and chromatin-accessibility data provides a more direct link between genetic susceptibility and cellular dysfunction in OA (Liu et al., 2018).

Mechanical stress may further influence MSCs through nuclear structural remodeling. Abnormal mechanical cues can be transmitted to the nucleus through integrins, the cytoskeleton, the nuclear envelope, and nuclear scaffold-associated structures, where they may alter chromatin organization and gene-expression states (Steward and Kelly, 2015; Saidova and Vorobjev, 2020). Although direct evidence from chondrogenic models of OA-derived MSCs remains limited, MSC mechanobiology studies support the view that mechanical inputs can be converted into nuclear and chromatin-level responses. For example, differentiation of MSCs can involve nuclear architectural remodeling, Lamin A/C redistribution, and increased heterochromatin formation. These observations suggest that OA-related altered functional state of MSCs may involve not only local enhancer or promoter changes but also broader reorganization of nuclear architecture, with consequences for differentiation direction and repair capacity (Heo et al., 2016; Ankam et al., 2018). Beyond local enhancer accessibility, three-dimensional chromatin organizers may also contribute to stem-cell fate regulation. The cohesin complex, composed of SMC1, SMC3, RAD21, and STAG subunits, regulates chromatin-loop structure, DNA repair, and gene transcription, thereby influencing stem-cell pluripotency, self-renewal, and differentiation (Xiang et al., 2025). Although direct evidence in OA-derived MSCs remains lacking, this mechanism supports the broader view that nuclear architecture can influence MSC fate.

In parallel, ATP-dependent chromatin remodeling complexes provide another physical mechanism for modulating chromatin accessibility. Switch/sucrose non-fermentable (SWI/SNF), imitation switch (ISWI), chromodomain helicase DNA-binding (CHD), and inositol requiring 80 (INO80) complexes use ATP hydrolysis to reposition, slide, evict, or exchange nucleosomes and histone variants, thereby regulating whether promoters, enhancers, and other regulatory elements remain accessible to transcriptional regulators (Eustermann et al., 2024). In MSC biology, BRG1/SMARCA4, the core ATPase of the SWI/SNF complex, has been linked to senescence regulation and stemness maintenance, suggesting that it may affect how MSCs respond transcriptionally to inflammatory, repair-related, and differentiation cues (Alessio et al., 2010). In OA, more direct evidence for remodeling factors still comes mainly from chondrocytes. BRG1 has been implicated in the protective effect of spermidine on OA cartilage, whereas SMARCA5 cooperates with RSF1 to activate glycolysis-related promoters and increase H3K27ac and transcriptional activity through an ATPase-dependent mechanism, thereby promoting pathological glycolysis and chondrocyte dysfunction (Mao et al., 2023; Xu et al., 2025).

These observations require caution when applied to MSCs. Direct evidence in OA-derived MSCs remains limited, and the currently available findings originate from different biological contexts. BRG1/SMARCA4 has primarily been implicated in stemness maintenance and senescence-associated transcriptional regulation in MSCs, whereas evidence for SMARCA5/RSF1-mediated chromatin remodeling currently comes mainly from OA chondrocytes, where it promotes glycolysis-related transcription and pathological metabolic reprogramming. Therefore, current evidence does not yet demonstrate that BRG1/SMARCA4 or SMARCA5 directly regulates impaired chondrogenesis in OA-derived MSCs. A more appropriate interpretation is that these remodeling factors represent candidate chromatin-state regulators that may influence OA-associated functional alterations of MSCs by affecting chromatin accessibility, enhancer activity, metabolism-related transcription, and cellular stress responses.

Chromatin accessibility, enhancer activity, nuclear architecture, and ATP-dependent remodeling are best treated as linked regulatory layers. Together, they help explain how repeated OA-related microenvironmental stress may contribute to reinforced but potentially reversible alterations in transcriptional programs and repair capacity of MSCs.

Direct validation in OA patient-derived MSCs, including synovial and bone marrow MSC populations, will require assay for transposase-accessible chromatin with sequencing (ATAC-seq), cleavage under targets and tagmentation (CUT&Tag), chromatin immunoprecipitation sequencing (ChIP-seq), single-cell transcriptomics, and targeted perturbation experiments. Such studies are needed to determine whether ATP-dependent remodeling factors directly regulate chondrogenic capacity, senescence, and paracrine function in OA-associated MSCs.

## 4 Epigenetic regulation and altered functional states of MSCs in OA

In OA joints, OA-associated functional alterations of MSCs should be interpreted as a shift in cellular state rather than as simple passive damage. Under physiological conditions, MSCs contribute to tissue homeostasis through self-renewal, multilineage differentiation, immunomodulation, and reparative paracrine activity. In contrast, OA-associated functional alterations refer to a shift toward a repair-unfavorable functional state characterized by reduced proliferative activity, impaired self-renewal capacity, weakened chondrogenic potential, altered immunomodulatory function, diminished regenerative capacity, and increased senescence-associated features. Under persistent pathological stimulation, these changes may develop as adaptive responses to the OA microenvironment. Importantly, they do not imply a complete or permanent loss of MSC identity or function, but rather reflect a dynamic and context-dependent cellular adaptation that is clinically less favorable for tissue repair and regeneration. Cellular senescence is one of the most evident features of this repair-unfavorable functional state. However, senescence-related changes should be interpreted cautiously because quiescence, insufficient activation, and true cellular senescence may share some superficial features, such as reduced proliferation (Liu et al., 2020; Weng et al., 2022; Dang et al., 2025). Evidence from OA-related joint tissues supports this view. For example, synovial MSCs isolated from OA patients have been reported to exhibit reduced colony-forming capacity, impaired proliferative activity, and decreased chondrogenic differentiation compared with MSCs from non-OA controls. Furthermore, clearance of senescent cells can partially restore their proliferative and chondrogenic capacities. MSCs derived from OA-associated synovial fluid may also display increased expression of senescence-associated markers together with reduced chondrogenic potential (Huang et al., 2020; Miura et al., 2022; Teti et al., 2023). These observations provide concrete examples of OA-associated functional alterations and indicate that the reduced reparative potential of MSCs is reflected in measurable declines in proliferative activity, clonogenicity, and chondrogenic differentiation.

Epigenetic dysregulation may help explain why some of these functional changes can be reinforced during chronic pathological exposure and may not be readily reversed under continued OA-associated stimulation. DNA methylation, histone modifications, and chromatin-state alterations can affect proliferation, senescence, lineage commitment, and immunomodulatory activity in MSCs (Zhao et al., 2022; Wang et al., 2020; Walewska et al., 2023). When inflammatory, oxidative, metabolic, and mechanical stresses persist, these epigenetic layers may gradually stabilize transcriptional programs that favor senescence and functional exhaustion over proliferation and chondrogenic repair. Chromatin remodeling adds a structural dimension to this process by changing nucleosome organization and chromatin accessibility. For example, the SWI/SNF core ATPase BRG1/SMARCA4 has been linked to senescence regulation in MSCs, suggesting that ATP-dependent remodeling may influence cell-cycle control, stemness maintenance, and senescence-associated transcriptional programs (Eustermann et al., 2024; Alessio et al., 2010).

A disease-associated regulatory state may therefore develop during chronic OA exposure. Whether this state should be defined as a true “memory-like” phenotype remains uncertain, but available evidence indicates that prolonged exposure to OA synovium, synovial fluid, or related inflammatory factors can leave MSCs in a repair-unfavorable condition. This condition is characterized by weaker chondrogenesis, stronger inflammatory responsiveness, and altered secretory behavior (Tan et al., 2023; Goldring and Marcu, 2012; Llewellyn et al., 2024). OA synovium- and synovial-fluid-derived factors can suppress chondrogenic differentiation of MSCs, while synovial MSCs from OA patients may themselves show pro-inflammatory and pro-degenerative tendencies (Fahy et al., 2014; Huang et al., 2020). These findings suggest that chronic pathological stimulation reshapes not only the extracellular environment but also the intrinsic regulatory state of MSCs.

Therefore, the functional quality of MSCs is as important as their quantity. MSCs isolated from different tissues of late-stage OA joints may retain some stemness-associated features, but their chondrogenic potential is unstable and does not clearly exceed that of age-matched bone marrow MSCs (Neybecker et al., 2020). This observation supports the view that disease exposure can leave a context-dependent imprint on the functional state of MSCs, which may persist under continued pathological stimulation but may also be modifiable if the microenvironment is improved. For OA regenerative therapy, the key issue is not only whether enough MSCs are present or delivered, but whether these cells retain a reparative phenotype. Restoring the endogenous activity of MSCs, reducing senescence-associated changes, and improving chondrogenic and immunomodulatory functions are therefore important goals for future OA repair strategies (Roberts et al., 2011; Sekelova et al., 2023; Jiang et al., 2023; Zhao et al., 2022).

## 5 Epigenetic regulation of paracrine function of MSCs and secretion of EVs

Repair mediated by MSCs in OA is not limited to their differentiation potential. Although MSCs can contribute to repair-related tissue formation, much of their therapeutic relevance is thought to arise from paracrine activity. Through soluble factors and extracellular vesicles (EVs), MSCs can influence inflammatory responses, matrix metabolism, and communication among joint-resident cells (Maumus et al., 2013; Dąbrowska et al., 2021; Kou et al., 2022). The epigenetic state of MSCs may affect both differentiation and paracrine output, but the evidence is not equally strong for these two aspects. Regulation of chondrogenesis by epigenetic mechanisms is relatively well supported, whereas direct evidence linking defined chromatin events to specific cargo loading into EVs remains limited (Walewska et al., 2023; Zhang et al., 2026).

For chondrogenic differentiation, the connection with epigenetic regulation is more direct. Enhancer activity, accessible chromatin states, and H3K27me3-related repression have all been associated with the establishment or inhibition of chondrogenic programs in MSCs. In human bone marrow MSCs, super-enhancer-associated lncRNA profiles change markedly during chondrogenic differentiation, suggesting that enhancer-level transcriptional regulation contributes to the formation of cartilage-related transcriptional programs (Jiang et al., 2021). Repressive H3K27me3-associated regulation is also involved in MSC fate transitions and in limiting access to cartilage-related gene expression programs (Ren et al., 2020). Several lncRNA–EZH2 mechanisms further illustrate this point. For example, lncRNA *MEG3* can recruit EZH2, promote H3K27me3 enrichment at the *TRIB2* promoter, and inhibit chondrogenic differentiation of synovium-derived MSCs (SMSCs). Similarly, lncRNA *CIR* suppresses *ATOH8* expression through EZH2-associated epigenetic repression and thereby weakens chondrogenic potential of MSCs (You et al., 2019; Liu et al., 2021). These findings support the idea that epigenetic states favoring cartilage-related gene expression may improve differentiation stability and repair potential of MSCs (Zhao et al., 2022).

The link between epigenetic regulation and EVs is more indirect. A more cautious interpretation is that the epigenetic state of MSCs shapes the overall secretory phenotype rather than directly determining the loading of individual cargo into EVs. Epigenetic regulation may change the types and quantities of microRNAs (miRNAs), long non-coding RNAs, proteins, and other bioactive molecules that can be packaged into EVs by altering transcription and secretion processes (Fatima et al., 2017; Martin-Rufino et al., 2019; Zhang et al., 2026). Therefore, chromatin remodeling should be considered as an upstream regulatory factor for EV-related functions. It can affect the cargo carried by EVs and their therapeutic activity by changing chromatin accessibility, enhancer output, inflammatory signals and metabolic status in donor MSCs (Martin-Rufino et al., 2019; Walewska et al., 2023; Zhang et al., 2026).

This distinction is important when discussing candidate chromatin regulators. HDAC subtypes, BRG1/SMARCA4, and SMARCA5 may influence transcriptional and secretory states of donor MSCs, but they should not be presented as direct switches controlling specific cargo loading into EVs (Alessio et al., 2010; Zhang et al., 2020; Xu et al., 2025). Current evidence more strongly supports the view that culture conditions, inflammatory priming, and preconditioning strategies reshape the donor-cell state, which then affects the miRNA composition and biological activity of EVs derived from MSCs. For example, exosomes derived from miR-140-5p-overexpressing synovial MSCs promoted cartilage matrix synthesis, increased COL2A1 and ACAN expression, and attenuated OA progression in experimental models (Tao et al., 2017). Consistent with this concept, inflammatory priming can influence miRNA detection profiles and donor-related variability in EVs derived from adipose-derived MSCs, whereas xeno-free culture and hypoxic preconditioning can alter miRNA cargo in EVs derived from bone marrow MSCs and improve their protective effects on inflammatory chondrocytes (Ragni et al., 2019; Palamà et al., 2023).

Preclinical OA studies generally support anti-inflammatory, matrix-protective, and repair-promoting effects of EVs derived from MSCs. Reported effects include reduced inflammatory and matrix-degrading mediators, enhanced cartilage matrix synthesis, and delayed cartilage degeneration (To et al., 2020; Liu et al., 2024; Wang et al., 2025). Clinical evidence, however, remains preliminary and less consistent. In a randomized, triple-blind, placebo-controlled trial, intra-articular placental EVs derived from MSCs showed an acceptable safety profile but did not improve symptomatic or magnetic resonance imaging (MRI) outcomes compared with placebo. In contrast, another translational study reported no obvious adverse events over 12 months after intra-articular administration of small EVs derived from umbilical cord MSCs (UC-MSC-sEVs), supporting early feasibility and safety (Bolandnazar et al., 2024; Figueroa-Valdés et al., 2025).

Therefore, the epigenetic state of MSCs should be linked to EVs through donor-cell transcriptional and secretory programs, not through a presumed one-to-one relationship between a chromatin event and a specific cargo profile of EVs. The mechanisms that determine selective cargo loading into EVs, release behavior, and downstream immunomodulatory or repair effects remain incompletely defined. Clarifying these links will be essential before epigenetic preconditioning or chromatin-targeted manipulation can be used reliably to optimize EV-based therapy for OA (Martin-Rufino et al., 2019; Boulestreau et al., 2021).

## 6 Therapeutic implications and translational prospects

The preceding sections primarily discuss epigenetic and chromatin-state alterations in endogenous MSC populations exposed to the OA microenvironment. In contrast, this section focuses on the translational implications of these findings, including strategies aimed at restoring endogenous MSC function as well as the therapeutic application of exogenously administered MSCs and MSC-derived EVs.

### 6.1 Major challenges in current OA and MSC-based therapy

The clinical translation of MSC-based OA therapy is limited by both disease complexity and product variability. OA is not driven by a single pathological pathway. Instead, inflammatory activity, increased or altered mechanical loading, metabolic imbalance, cellular senescence, and extracellular matrix remodeling interact within the joint. This multifactorial disease environment helps explain why single-target interventions or simple supplementation of MSCs often fail to produce durable therapeutic benefit (Yao et al., 2023; Dang et al., 2025).

A second challenge comes from the biological heterogeneity of MSCs themselves. Although MSCs have reparative potential, their therapeutic performance can vary according to tissue source, donor age, cellular subpopulation, expansion history, and the microenvironment encountered after administration. In OA joints, local inflammatory stress, senescence-associated signals, altered matrix conditions, and impaired differentiation cues may further reduce survival, functional secretion, and repair stability of MSCs. These factors make it difficult to achieve consistent efficacy across patients and preparations (Zha et al., 2021; Sun et al., 2023; Santolini et al., 2025).

Therapies based on EVs derived from MSCs face related but distinct translational barriers. Compared with whole-cell therapy, EVs may offer advantages in storage, delivery, and safety control, but their mechanisms of action remain incompletely defined. Preparation methods, isolation procedures, cargo characterization, potency assays, dosing standards, and indication stratification still vary widely across studies. Without clearer standards for production and functional evaluation, even promising preclinical findings are difficult to translate into reproducible clinical outcomes (Dąbrowska et al., 2021; Martin-Rufino et al., 2019; D’Arrigo et al., 2024; González-Rodríguez et al., 2025).

Therefore, the key obstacle is not whether MSCs or EVs can exert repair-related effects, but whether their biological activity can be made predictable, stable, and matched to the disease status of individual OA patients. This challenge provides a rationale for examining epigenetic and chromatin-state regulation, because these mechanisms may help explain why MSCs lose reparative capacity in the OA microenvironment and how their functional state might be optimized before clinical application.

### 6.2 How epigenetic regulation deepens mechanistic understanding

Epigenetic regulation helps explain why MSCs may show inconsistent therapeutic effects in OA. The problem is unlikely to be limited to insufficient cell number or weak local stimulation. Under chronic inflammatory, mechanical, oxidative, metabolic, and senescence-associated stress, MSCs may gradually acquire a regulatory state that is less responsive to repair-promoting signals and less capable of maintaining stable chondrogenic or immunomodulatory functions (Franzen et al., 2016; Liu et al., 2020; Sun et al., 2023).

This perspective shifts attention from acute signaling responses to the mechanisms that maintain or reinforce repair-unfavorable functional programs during chronic OA exposure. Rather than implying a permanently dysfunctional state, this framework emphasizes how sustained pathological stimuli may continuously shape MSC behavior through dynamic and potentially reversible regulatory processes. DNA methylation, histone modifications, non-coding RNAs, and RNA modifications can alter the transcriptional programs of MSCs, while chromatin accessibility, enhancer activity, and ATP-dependent remodeling determine whether these programs remain accessible, restricted, or reconfigured. In this way, OA-related stress may be converted into sustained but potentially reversible transcriptional programs that move MSCs away from a repair-supportive phenotype (Ren et al., 2020; Zha et al., 2021; Walewska et al., 2023; Eustermann et al., 2024).

Epigenetic regulation therefore provides a mechanistic bridge between the pathological joint microenvironment and unstable MSC-based repair. It suggests that OA is not only a failure of damaged joint tissues to regenerate, but also a process in which local repair-associated cells are progressively remodeled by disease-related stress. Within this framework, HDAC subtypes and SWI/SNF/ISWI-related chromatin-state regulators are useful molecular nodes for studying how repeated microenvironmental stimulation may reinforce altered transcriptional programs without necessarily producing irreversible cellular states. However, these nodes should be interpreted according to evidence source and cell type, because findings from chondrocytes or general MSC models cannot automatically be applied to OA-derived MSCs.

### 6.3 Potential therapeutic advantages of epigenetic regulation

The therapeutic value of epigenetic regulation lies in its ability to modify cellular state rather than simply increase cell number. In OA, the repair capacity of MSCs is weakened by senescence, inflammatory stimulation, and epigenetic dysregulation. These factors reduce proliferation, self-renewal, chondrogenic differentiation, and immunomodulatory activity (Tan et al., 2023; Zhang et al., 2026; Cheng et al., 2023). If OA-associated altered functional state of MSCs is partly maintained by reversible epigenetic states, then rejuvenation strategies, metabolic adjustment, hypoxic preconditioning, or targeted modulation of key epigenetic nodes may help restore chondrogenic potential and delay senescence-related phenotypes. For a chronic local disease such as OA, local and controllable epigenetic modulation may be more feasible than broad systemic intervention (Tan et al., 2023; Smith et al., 2023).

Epigenetic regulation also supports the rationale for microenvironmental engineering. Introducing MSCs into a hostile OA joint without changing the surrounding niche is unlikely to produce stable repair. Synovial inflammation, senescence-associated stress, hypoxia, hyperglycemia, hyperlipidemia, and altered matrix conditions can continuously influence MSC survival, fate decisions, and therapeutic performance (Zhang H. et al., 2024; Sun et al., 2023). These factors may not only trigger short-term cellular responses but also reshape chromatin status, DNA methylation patterns, and transcriptional programs over time. Therefore, biomaterials, scaffolds, and local microenvironmental control should be designed not only to deliver MSCs but also to create a niche that preserves their reparative phenotype. In this context, improving the local environment may be as important as increasing the number of administered cells (Chen, 2022; Liang et al., 2023).

Therapies based on EVs provide another translational direction. Compared with whole-cell therapy using MSCs, EVs derived from MSCs may offer advantages in storage, delivery, and safety control while retaining part of the paracrine, immunomodulatory, and repair-supportive effects of donor cells (Kou et al., 2022; Li et al., 2025). However, EV function is strongly influenced by the state of donor MSCs. Epigenetic state, inflammatory priming, oxygen tension, metabolic conditions, and culture system may all affect the secretome and cargo composition of EVs, especially miRNAs and other non-coding RNAs (Martin-Rufino et al., 2019; Lara-Barba et al., 2021). Hypoxic preconditioning, for example, can reshape the miRNA composition of EVs derived from MSCs and enhance cartilage-repair, anti-apoptotic, and pro-migratory/pro-proliferative effects, whereas different culture and preconditioning systems can alter anti-inflammatory cargo and therapeutic activity (Zhang et al., 2022; Palamà et al., 2023). Thus, optimization of EVs may be achieved either by preconditioning MSCs before EVs collection or by engineering EVs directly to improve targeting and biological activity (Liu et al., 2023; Li et al., 2025).

Targeting chromatin state may further expand therapeutic options, but this strategy requires caution. One possible approach is to regulate histone acetylation through HDAC inhibitors or subtype-selective HDAC modulation, thereby influencing chromatin accessibility and transcriptional programs related to cartilage homeostasis. Another approach is to target ATP-dependent remodeling factors, such as SWI/SNF or ISWI complexes, to modify nucleosome positioning and regulatory-element accessibility. These strategies are conceptually attractive because epigenetic and chromatin-state regulation is dynamic and potentially reversible. However, the same chromatin regulator may have context-dependent or subtype-specific effects, and tissue-specific delivery remains difficult. Therefore, chromatin-targeted strategies should be developed together with patient-derived MSCs/chondrocyte models, multi-omics profiling, and functional validation to identify which targets are both effective and safe for stratified OA treatment (Zhang et al., 2020; Barter et al., 2022; Gu et al., 2024; Xu et al., 2025).

### 6.4 Current limitations and translational challenges

Despite its mechanistic appeal, epigenetic regulation is not yet a straightforward therapeutic solution for OA. A major difficulty is that the epigenetic network in MSCs does not operate through isolated marks. DNA methylation, histone modifications, non-coding RNAs, RNA modifications, chromatin accessibility, and ATP-dependent remodeling influence one another. Their functional outcome depends on cell type, disease stage, local microenvironment, and the timing of stimulation. For this reason, a change in one epigenetic mark cannot be assumed to produce a predictable change in behavior of MSCs without considering the broader regulatory context (Tan et al., 2023; Smith et al., 2023; Walewska et al., 2023).

Another major obstacle is specificity. Many epigenetic interventions affect a wide range of regulatory processes, rather than a single disease-related pathway. If used directly *in vivo*, these interventions may alter the transcription of non-target cells, trigger unexpected reprogramming, or pose long-term safety risks. These risks are of particular concern in OA, as the joint contains a variety of interacting tissues, including cartilage, synovium, subchondral bone, immune cells, and local progenitor cell populations. Therefore, epigenetic therapy for OA will require not only effective molecular targets but also delivery systems and dosing strategies that restrict the intervention to appropriate cells and disease stages (Zhao et al., 2022; Zhang et al., 2026).

The current evidence base also remains uneven. Many conclusions are derived from *in vitro* experiments, animal models, non-OA-derived cells, or chondrocyte studies rather than patient-derived OA MSCs. This limits causal interpretation. For example, HDAC subtypes mainly belong to the histone-modification regulatory layer, whereas BRG1/SMARCA4 and SMARCA5 are ATP-dependent chromatin remodeling factors. These molecules should therefore be discussed according to both regulatory hierarchy and evidence source (Zhang et al., 2020; Eustermann et al., 2024). BRG1/SMARCA4 has been linked to MSCs senescence, and SMARCA5/RSF1 has been associated with pathological glycolysis in OA chondrocytes. However, these observations do not yet prove that the same mechanisms directly control chondrogenic differentiation, senescence, or EVs-related function in OA-derived MSCs (Alessio et al., 2010; Xu et al., 2025). Similarly, cohesin-mediated three-dimensional chromatin organization may represent an upstream regulatory layer of stem-cell fate determination, but direct evidence linking this mechanism to chondrogenic differentiation, senescence, or EV-related function in OA-derived MSCs remains lacking.

The same caution applies to EVs derived from MSCs. Although EVs and engineered EVs are promising for OA treatment, the mechanisms connecting donor-cell epigenetic state with cargo loading, release behavior, tissue targeting, and therapeutic output remain incompletely defined. At present, it is safer to view epigenetic or chromatin-state regulation as an upstream influence on the donor-cell secretory program rather than as a precise cargo-loading mechanism. More direct studies are needed to determine whether specific epigenetic interventions can reproducibly improve potency of EVs without increasing heterogeneity or safety risks (Martin-Rufino et al., 2019; Zhang et al., 2026; Lara-Barba et al., 2021; Mussin et al., 2025).

Patient and product heterogeneity further complicate translation. OA patients differ in age, disease duration, inflammatory burden, metabolic status, joint microenvironment, and extent of joint involvement. MSCs and EVs derived from MSCs also vary according to tissue source, donor characteristics, cell subpopulations, culture systems, and preconditioning protocols. These variables can influence biological activity, batch-to-batch consistency, and therapeutic efficacy (Zhang H. et al., 2024; Zha et al., 2021; Samsonraj et al., 2017; Tarasova et al., 2025). Therefore, therapies based on epigenetic regulation should not rely on a single uniform model. They will need patient stratification, standardized manufacturing of MSCs and EVs, validated potency assays, rational dose standards, appropriate clinical endpoints, and long-term safety follow-up (Maumus et al., 2020; Aziziyan et al., 2026).

In general, the challenge at the translational level is not whether there is a biological association between epigenetic regulation and OA-associated functional alterations of MSCs, but whether it can be precisely controlled to improve the stability of treatment. Clinical-grade MSC-derived EVs have initially met the feasibility and safety requirements, but the current progress cannot support their large-scale clinical application (Figueroa-Valdés et al., 2025). Before epigenetic or chromatin-targeted strategies can move toward routine OA therapy, future studies must define causal mechanisms in OA patient-derived MSCs, reduce product heterogeneity, and establish safety, potency, and efficacy standards that are reproducible across centers (Li et al., 2025; González-Rodríguez et al., 2025; Aziziyan et al., 2026) (Table 2).

**TABLE 2:** Candidate OA regenerative therapy strategies linked to epigenetic and chromatin-state regulation of MSCs and their major limitations.

Strategy	Condensed rationale/expected benefit	Major limitation	Representative supporting references
MSC rejuvenation and epigenetic preconditioning (e.g., hypoxia, metabolic adjustment, inflammatory priming)	May partially restore repair-supportive MSC states by reducing senescence-associated features and improving chondrogenic, immunomodulatory, or paracrine activity	Effects depend on donor source, disease stage, culture protocol, and whether true senescence is distinguished from quiescence or insufficient activation	Liu et al. (2020); Cheng et al. (2023); Smith et al. (2023); Tan et al. (2023); Ragni et al. (2019); Palamà et al. (2023)
Microenvironmental engineering (e.g., biomaterials, scaffolds, local niche modulation)	May protect administered or endogenous MSCs from the hostile OA niche and preserve reparative phenotypes through local control of inflammatory, mechanical, metabolic, and matrix-related cues	Delivery design, mechanical matching, tissue specificity, durability, and long-term safety remain unresolved	Chen (2022); Liang et al. (2023); Sun et al. (2023); Zhang H. et al. (2024)
MSC-derived EV optimization and engineering (e.g., hypoxic preconditioning, xeno-free culture, miR-140-enriched EVs)	May enhance anti-inflammatory, matrix-protective, and cartilage-repair effects by improving donor-cell state or modifying EV cargo composition	EV cargo loading, potency assays, dosing, manufacturing standardization, and clinical efficacy remain incompletely defined	Tao et al. (2017); Martin-Rufino et al. (2019); Ragni et al. (2019); Dąbrowska et al. (2021); Kou et al. (2022); Palamà et al. (2023); Bolandnazar et al. (2024); Figueroa-Valdés et al. (2025); Li et al. (2025)
Targeted epigenetic or chromatin-state modulation (e.g., HDAC, EZH2, BRG1/SMARCA4, SMARCA5-related pathways)	May regulate histone marks, chromatin accessibility, enhancer activity, nucleosome positioning, and transcriptional programs linked to MSC dysfunction or OA-related cellular stress	Most evidence remains preclinical or derived from chondrocytes, non-OA MSCs, or general MSC models; off-target and subtype-specific effects require validation in OA-derived MSCs	Alessio et al. (2010); Ren et al. (2020); Liu et al. (2021); You et al. (2019) Zhang et al. (2020); Barter et al. (2022); Gu et al. (2024); Xu et al. (2025)

Abbreviations are defined in the main text. The listed strategies represent candidate translational approaches rather than validated clinical interventions.

## 7 Discussion and conclusion

OA-associated repair failure cannot be explained only by cartilage loss, inflammation, or an insufficient number of reparative cells. A central issue is that the OA joint repeatedly exposes local MSCs to inflammatory, mechanical, metabolic, oxidative, and senescence-related stress. These pressures may be incorporated into MSC regulatory programs in a dynamic manner, with some responses being transient and adaptive, whereas others may be reinforced by epigenetic and chromatin-state regulation under sustained pathological stimulation. These regulatory mechanisms should not be considered as isolated events but rather as interconnected layers of a coordinated epigenetic network. In the OA microenvironment, persistent inflammatory, mechanical, metabolic, and senescence-associated stimuli may induce changes in DNA methylation, repressive or activating histone modifications, chromatin accessibility, and ncRNA/miRNA expression. These alterations interact with one another to influence enhancer activity, transcription-factor binding, and nucleosome organization, ultimately reshaping gene-expression programs involved in stemness maintenance, chondrogenic differentiation, senescence, and paracrine function. Collectively, these processes may transiently or persistently shift MSCs from a repair-supportive phenotype toward an OA-associated functional state. Rather than representing a permanent loss of MSC identity or a complete loss of function, this state is more appropriately interpreted as a dynamic cellular adaptation to the pathological microenvironment. However, compared with physiological conditions, the reparative, regenerative, and chondrogenic capacities of MSCs may become substantially compromised. Therefore, future studies should distinguish MSC-intrinsic senescence from senescence-associated signals derived from surrounding OA joint cells and should further separate true senescence from quiescence or failed MSC activation.

Chromatin remodeling should therefore be positioned as the structural component of the broader epigenetic network. Its importance lies in converting regulatory marks and complex recruitment into changes in chromatin accessibility, enhancer use, and nucleosome organization. This interpretation also helps explain why simple cell supplementation may produce unstable outcomes in OA. Even when MSCs are present or administered, a hostile joint niche may continue to push them toward a repair-unfavorable state. In this context, HDAC subtypes, BRG1/SMARCA4, and SMARCA5 are best regarded as candidate regulatory nodes rather than fully established therapeutic targets. Their relevance must be interpreted according to cell type, disease context, and evidence source, especially because much of the current evidence still comes from chondrocytes, non-OA-derived MSCs, or general senescence models.

The next priority is to establish causal evidence in OA patient-derived MSCs. Multi-omics profiling, including ATAC-seq, CUT&Tag, ChIP-seq, single-cell transcriptomics, and metabolomics, needs to be combined with functional perturbation to determine which chromatin and epigenetic changes are drivers rather than bystanders. For translation, the key challenge is not only to identify promising epigenetic targets but also to control their effects with sufficient precision. Patient stratification, standardized manufacturing of MSCs and EVs, validated potency assays, long-term safety assessment, and reproducible efficacy evaluation will be essential before epigenetic or chromatin-targeted regenerative strategies can move toward clinical application in OA.

## Data Availability

The original contributions presented in the study are included in the article/supplementary material, further inquiries can be directed to the corresponding authors.
